# Effects of Flunarizine and Lutein on Atezolizumab-Associated Ovarian Alterations in Rats

**DOI:** 10.3390/ijms27219862

**Published:** 2026-11-03

**Authors:** Ummugulsum Esenkaya, Esra Tuba Sezgin, Sevinc Haciyeva, Bahadir Suleyman, Mustafa Ozkaraca, Ali Gungor, Taha Abdulkadir Coban, Halis Suleyman

**Affiliations:** 1 Department of Obstetrics and Gynecology, Konya City Hospital, University of Health Sciences, Konya 42020, Türkiye; ummugulsum.esenkaya@sbu.edu.tr; 2 Anesthesia Program, Vocational School of Health Services, Erzincan Binali Yıldırım University, Erzincan 24100, Türkiye; esra.demir@erzincan.edu.tr; 3 Department of Pathological Physiology, Azerbaijan Medical University, Baku AZ 1022, Azerbaijan; sevinchaciyeva890@gmail.com; 4 Department of Pharmacology, Faculty of Medicine, Erzincan Binali Yıldırım University, Erzincan 24100, Türkiye; bsuleyman@erzincan.edu.tr; 5 Department of Pathology, Faculty of Veterinary Medicine, Sivas Cumhuriyet University, Sivas 58140, Türkiye; mustafaozkaraca@cumhuriyet.edu.tr; 6 Laboratory and Veterinary Health Program, Vocational School of Health Services, Osmaniye Korkut Ata University, Osmaniye 80000, Türkiye; aligungor@osmaniye.edu.tr; 7 Department of Medical Biochemistry, Faculty of Medicine, Erzincan Binali Yıldırım University, Erzincan 24100, Türkiye; acoban@erzincan.edu.tr

**Keywords:** ovarian alterations, oxidative stress, lipid peroxidation, inflammatory cytokines, histopathology, immunofluorescence, experimental rat model

## Abstract

Immune checkpoint inhibitors (ICIs), including atezolizumab, enhance antitumor immune responses by interfering with inhibitory immune checkpoint signaling. Atezolizumab targets programmed death-ligand 1 (PD-L1), thereby disrupting its interaction with programmed death-1 (PD-1). Although ICIs have improved cancer treatment outcomes, their potential associations with ovarian alterations remain insufficiently understood. This study aimed to compare ovarian biochemical, hormonal, histopathological, and immunofluorescence outcomes following the co-administration of flunarizine or lutein with atezolizumab in rats. Twenty-four female Wistar rats were randomly assigned to four groups (*n* = 6 per group): healthy control (HG), atezolizumab (ATZ), flunarizine plus atezolizumab (FUAZ), and lutein plus atezolizumab (LTAZ). Ovarian oxidative stress markers (MDA, tGSH, SOD, and CAT), inflammatory cytokines (TNF-α and IL-1β), serum anti-Müllerian hormone (AMH) and prolactin levels, histopathological alterations, and immunofluorescence intensities of COX-1, COX-2, IL-1β, and IL-6 were evaluated. Compared with HG, the ATZ group exhibited increased MDA, TNF-α, and IL-1β levels and decreased tGSH, SOD, CAT, and serum AMH levels (*p* < 0.05). Histopathological examination revealed more pronounced follicular degeneration, parenchymal edema, and hyperemia in the ATZ group, whereas these alterations were milder in the FUAZ group and moderate in the LTAZ group. Immunofluorescence analysis demonstrated reduced COX-1 and increased COX-2, IL-1β, and IL-6 intensities in the ATZ group compared with HG. Both co-administration groups showed higher COX-1 and lower COX-2, IL-1β, and IL-6 intensities than the ATZ group. Several biochemical, histopathological, and immunofluorescence outcomes in the FUAZ group were closer to HG values than those in the LTAZ group. Serum prolactin levels differed significantly only between the ATZ and FUAZ groups in pairwise comparisons. Atezolizumab administration was associated with ovarian oxidative, inflammatory, and histopathological alterations. Co-administration of flunarizine or lutein was associated with differences in several evaluated parameters, with more favorable findings observed in the FUAZ group at the selected doses. However, the absence of appropriate antibody controls and adjunct-only groups limits causal attribution and precludes conclusions regarding independent protective efficacy.

## 1. Introduction

Atezolizumab is a humanized monoclonal antibody that specifically binds to programmed death-ligand 1 (PD-L1) and sterically blocks its interaction with programmed death-1 (PD-1), thereby disrupting PD-1/PD-L1-mediated inhibitory signaling, as demonstrated by structural analyses of the atezolizumab–PD-L1 complex [1,2]. Atezolizumab has demonstrated clinical efficacy across multiple cancer types and represents an important immune checkpoint inhibitor in cancer therapy [3]. Although previous experimental studies have demonstrated cross-reactivity of atezolizumab with rat PD-L1 [4], direct in vivo target binding and functional PD-L1 blockade were not confirmed in the present study. Therefore, the observed ovarian alterations should not be unequivocally attributed to PD-L1 checkpoint inhibition. Furthermore, the absence of an isotype- or formulation-matched intraperitoneal control group precludes the exclusion of potential contributions from nonspecific antibody effects, formulation excipients, or injection-related stress. Accordingly, the findings should be interpreted as atezolizumab administration-associated ovarian alterations of uncertain mechanism rather than definitive evidence of PD-L1 blockade-mediated reproductive toxicity. These results neither establish the efficacy of interventions targeting PD-L1 blockade-mediated injury nor elucidate the mechanisms underlying immune checkpoint inhibitor-associated toxicity.

Immune checkpoint inhibitors (ICIs) have improved treatment responses and survival outcomes in several malignancies. However, therapeutic resistance and immune-related adverse events remain important limitations [5]. Atezolizumab, a monoclonal antibody targeting programmed death-ligand 1 (PD-L1), is used in the treatment of several cancers, including urothelial carcinoma and non-small cell lung cancer. Despite its therapeutic benefits, atezolizumab has been associated with adverse effects involving multiple organ systems [6] and ICI-related adverse effects involving the reproductive system have also been reported [7]. Evidence regarding the reproductive effects of immune checkpoint inhibitors remains limited because reproductive outcomes have not been systematically investigated. Further studies using species-appropriate experimental models, validated ovarian assessments, and mechanistic investigations are needed to clarify these effects. Although rare, ovarian failure has been documented, particularly in association with immune-related endocrine toxicities [8]. Ovarian insufficiency may manifest as menstrual irregularities, infertility, and estrogen deficiency-related symptoms [9]. Depending on the underlying etiology, ovarian dysfunction may be associated with follicular depletion or impaired follicular development [10]. Management strategies include hormone replacement therapy, fertility counseling, and, when appropriate, fertility preservation approaches [9,10]. Experimental studies have also reported changes in ovarian inflammatory and oxidative stress-related parameters [11]. Oxidative stress and inflammatory responses may contribute to ovarian tissue alterations through increased reactive oxygen species (ROS) production, lipid peroxidation, and proinflammatory cytokine signaling. These processes may disrupt cellular homeostasis and contribute to follicular and stromal damage [12]. However, the specific molecular pathways underlying the ovarian alterations observed in the present experimental model remain unclear. Therefore, these observations are presented as background information rather than evidence of a specific mechanism underlying the observed ovarian alterations.

Lutein is a naturally occurring bioactive xanthophyll carotenoid commonly found in the human diet [13,14,15]. It exhibits antioxidant, anti-inflammatory, and antiviral properties [16]. Its antioxidant effects are associated with the neutralization of reactive oxygen species and reduction in lipid peroxidation, while its anti-inflammatory activity involves the modulation of inflammatory signaling pathways and pro-inflammatory cytokine production.

Flunarizine is a selective Ca^2+^ channel blocker [17] that has also been reported to inhibit Na^+^ channels [18]. Its inhibition of T-type Ca^2+^ and Na^+^ channels has been associated with antiangiogenic effects [19]. In addition, flunarizine has antioxidant properties, has been reported to protect against mitochondrial damage and increase cellular ATP levels [20], and also exhibits anti-inflammatory effects [21].

The reported antioxidant and anti-inflammatory properties of lutein and flunarizine provided the rationale for evaluating these agents as co-treatments in the present experimental model. To the best of our knowledge, no previous experimental study has simultaneously evaluated flunarizine and lutein in the context of atezolizumab-associated ovarian alterations. The novelty of the present study lies in the comprehensive and comparative evaluation of ovarian oxidative stress, inflammatory responses, hormonal parameters, histopathological changes, and immunofluorescence findings within the same experimental model. Accordingly, the present study aimed to compare the biochemical, histopathological, hormonal, and immunofluorescence outcomes observed following the co-administration of flunarizine or lutein with atezolizumab at doses previously investigated in experimental studies.

## 2. Results

### 2.1. Histopathological Findings

**Figure 1:**
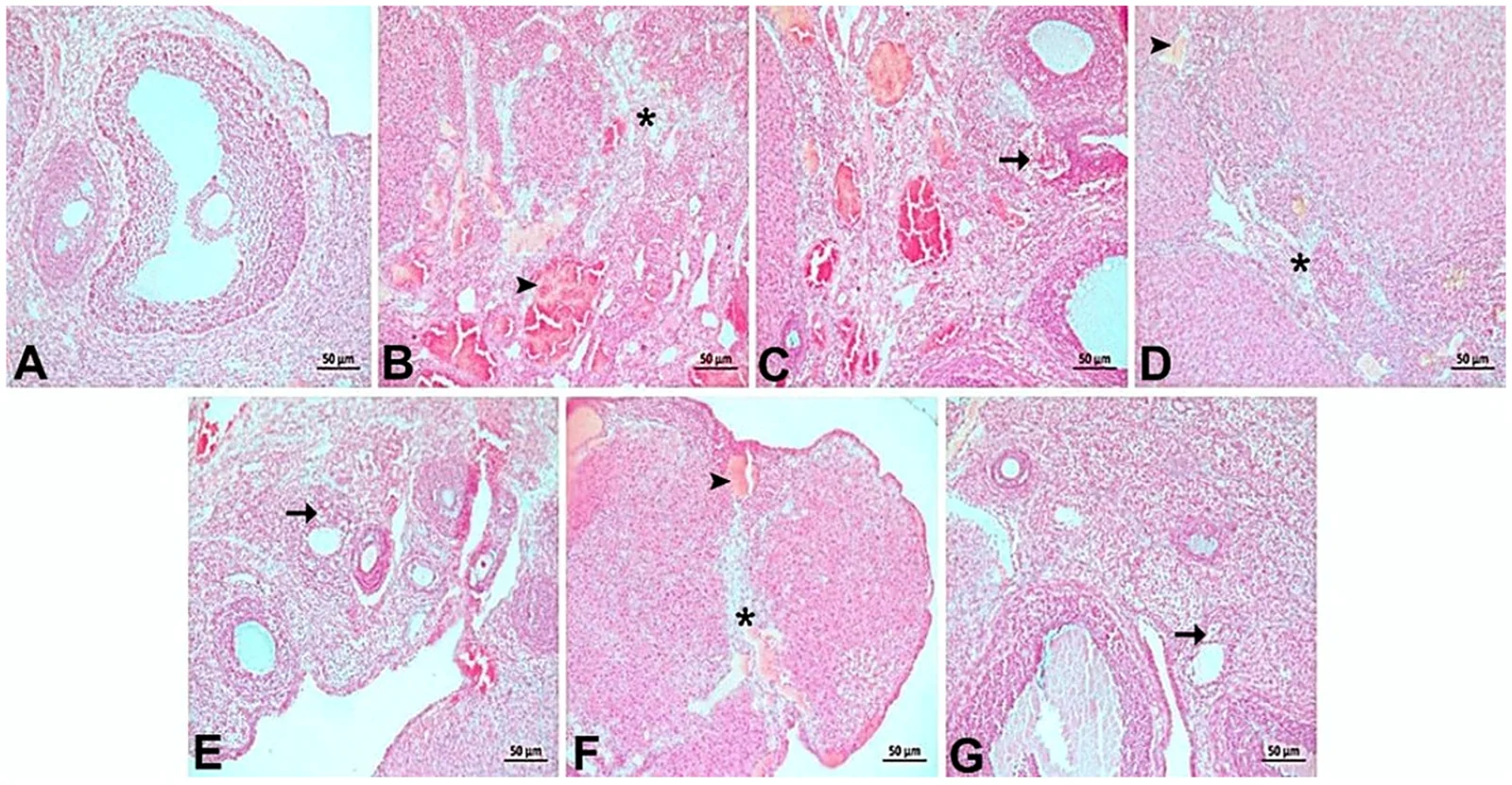
Representative histopathological images of ovarian tissues. (**A**) HG showing normal histological appearance. (**B**,**C**) ATZ group showing intense follicular degeneration (→), edema (*), and hyperemia (►). (**D**,**E**) FUAZ group showing mild follicular degeneration (→), edema (*), and hyperemia (►). (**F**,**G**) LTAZ group showing moderate follicular degeneration (→), edema (*), and hyperemia (►). H&E staining, ×400; scale bar = 50 μm.

Histopathological examination revealed significant differences among the experimental groups in terms of follicular degeneration, parenchymal edema, and vascular hyperemia (Table 1 and Figure 1; *p* < 0.05). The healthy control (HG) group exhibited preserved ovarian histological architecture, with no evidence of follicular degeneration, edema, or hyperemia. In contrast, the atezolizumab-treated (ATZ) group showed pronounced ovarian histopathological alterations, characterized by severe follicular degeneration, marked parenchymal edema, and prominent vascular hyperemia. These alterations were reflected in the higher histopathological scores observed in the ATZ group. In the flunarizine plus atezolizumab (FUAZ) group, follicular degeneration was mild, accompanied by relatively limited parenchymal edema and vascular hyperemia. The lutein plus atezolizumab (LTAZ) group exhibited moderate follicular degeneration, edema, and hyperemia, indicating more pronounced histopathological alterations than those observed in the FUAZ group. Statistical analysis demonstrated that the histopathological scores were significantly lower in the FUAZ group than in the LTAZ group (Table 1). Overall, the histopathological findings indicated that flunarizine co-administration was associated with less severe ovarian morphological alterations than lutein co-administration at the investigated doses.

### 2.2. Biochemical Results

**Figure 2:**
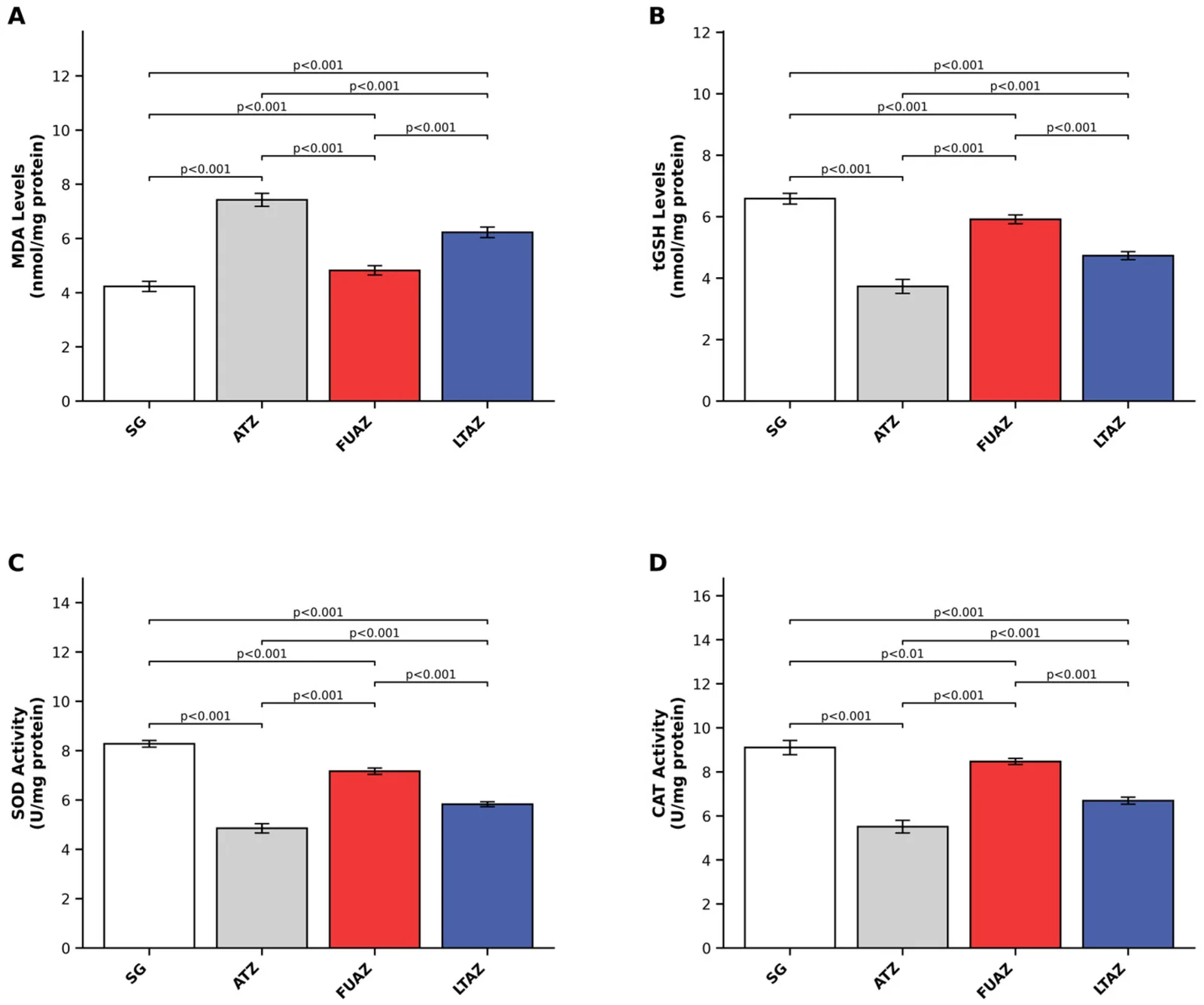
Effects of flunarizine and lutein on ovarian tissue MDA, tGSH, SOD, and CAT levels in atezolizumab-treated rats. (**A**) MDA, (**B**) tGSH, (**C**) SOD, and (**D**) CAT levels in ovarian tissues. Data are presented as mean ± SD *(n* = 6 per group). Statistically significant differences between groups are indicated by horizontal brackets and corresponding *p*-values, as determined by one-way ANOVA followed by Tukey’s HSD post hoc test (*p* < 0.05).

MDA levels were highest in the ATZ group (7.42 ± 0.24) and lowest in the HG (4.23 ± 0.19), with significantly lower levels in FUAZ (4.82 ± 0.17) and LTAZ (6.23 ± 0.20) than in ATZ (FUAZ < LTAZ). Conversely, tGSH levels were lowest in ATZ (3.73 ± 0.23) and highest in HG (6.59 ± 0.17), with significantly higher levels in FUAZ (5.91 ± 0.14) and LTAZ (4.73 ± 0.13) than in ATZ (FUAZ > LTAZ). Similarly, SOD and CAT activities were significantly higher in FUAZ and LTAZ than in ATZ, with higher values in FUAZ than in LTAZ (Figure 2 and Supplementary Table S1).

### 2.3. Results of Pro-Inflammatory Cytokines

**Figure 3:**
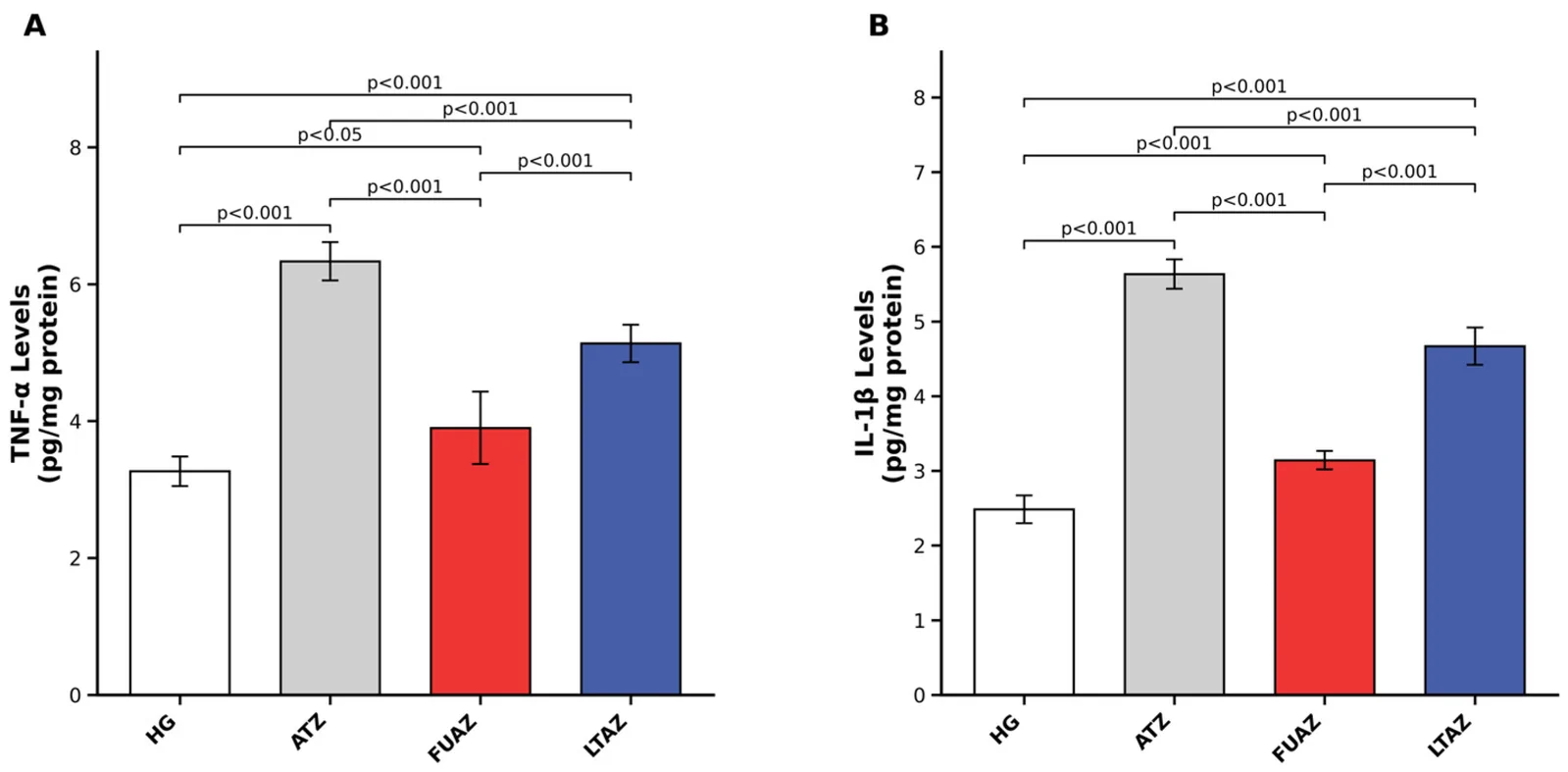
Effects of flunarizine and lutein on ovarian tissue TNF-α and IL-1β levels in atezolizumab-treated rats. (**A**) TNF-α, (**B**) IL-1β levels in ovarian tissues. Data are presented as mean ± SD (*n* = 6 per group). Statistically significant differences between groups are indicated by horizontal brackets and corresponding *p*-values, as determined by one-way ANOVA followed by Tukey’s HSD post hoc test (*p* < 0.05).

TNF-α levels were significantly higher in the ATZ group than in the HG and significantly lower in the FUAZ and LTAZ groups than in the ATZ group. TNF-α levels were also significantly lower in the FUAZ group than in the LTAZ group (all *p* < 0.001). Similarly, IL-1β levels were significantly higher in the ATZ group than in the HG and significantly lower in both the FUAZ and LTAZ groups than in the ATZ group. IL-1β levels were also significantly lower in the FUAZ group than in the LTAZ group (all *p* < 0.001) (Figure 3 and Supplementary Table S2).

### 2.4. Serum Hormone Levels

#### 2.4.1. Serum Prolactin Levels

**Figure 4:**
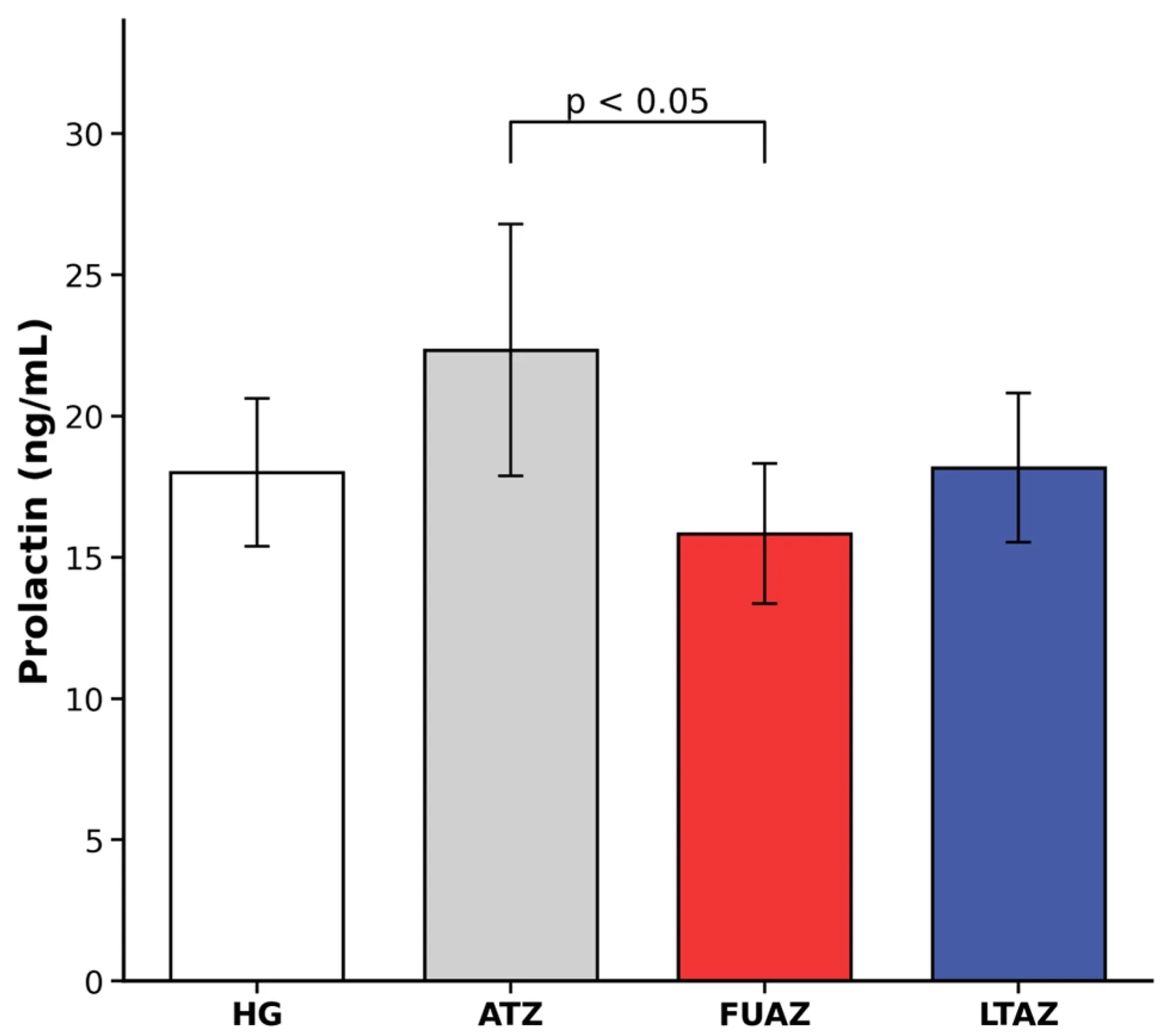
Effects of flunarizine and lutein on serum prolactin levels in atezolizumab-treated rats. Data are presented as mean ± SD (*n* = 6 per group). Statistically significant differences between groups are indicated by horizontal brackets and corresponding *p*-values, as determined by one-way ANOVA followed by Tukey’s HSD post hoc test (*p* < 0.05).

As shown in Figure 4 and Supplementary Table S3, serum prolactin levels differed significantly among the experimental groups (one-way ANOVA, *p* < 0.05). The highest mean prolactin level was observed in the ATZ group (22.33 ± 4.46 ng/mL), whereas the lowest was recorded in the FUAZ group (15.83 ± 2.48 ng/mL). Tukey’s HSD post hoc test revealed a significant difference between the ATZ and FUAZ groups (*p* < 0.05). No significant differences were observed in the remaining pairwise comparisons (*p* > 0.05). Notably, prolactin levels did not differ significantly between the HG and ATZ groups, indicating that the present findings do not provide sufficient evidence of an atezolizumab-associated increase in serum prolactin.

#### 2.4.2. Serum AMH Levels

**Figure 5:**
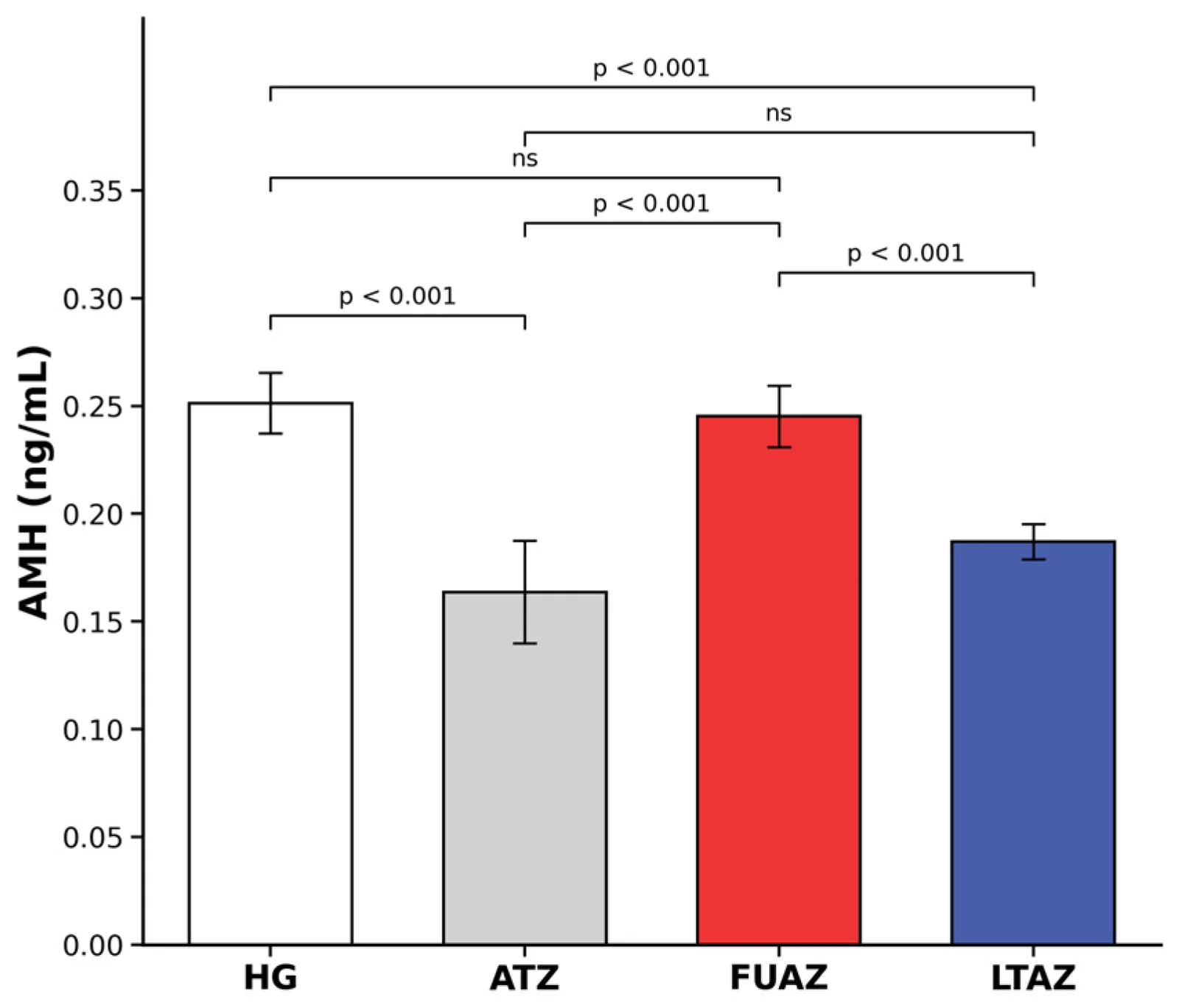
Effects of flunarizine and lutein on serum AMH levels in atezolizumab-treated rats. Data are presented as mean ± SD (*n* = 6 per group). Statistically significant differences between groups are indicated by horizontal brackets and corresponding *p*-values, as determined by one-way ANOVA followed by Tukey’s HSD post hoc test (*p* < 0.05) (ns, not statistically significant (*p* > 0.05)).

As shown in Figure 5 and Supplementary Table S4, serum AMH levels were significantly lower in the ATZ and LTAZ groups than in the HG and FUAZ groups (*p* < 0.05). No statistically significant differences were observed between the HG and FUAZ groups or between the ATZ and LTAZ groups (*p* > 0.05). However, because the AMH assay was not independently validated for rat serum, these findings should be considered exploratory and should not be interpreted as definitive evidence of changes in ovarian reserve.

### 2.5. Double Immunofluorescence Findings

**Figure 6:**
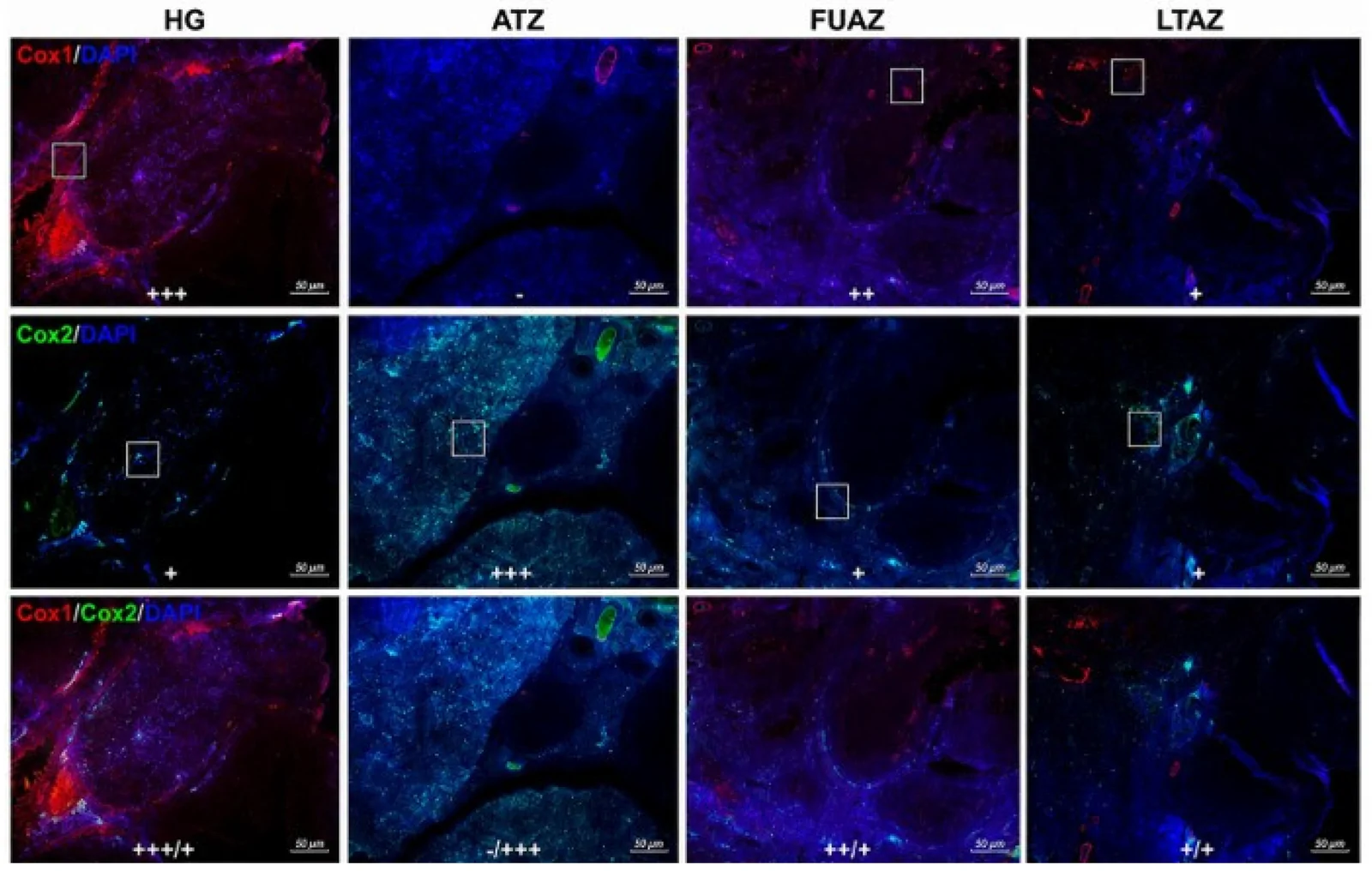
COX-1 immunopositivity was intense in the HG, absent in the ATZ group, moderate in the FUAZ group, and mild in the LTAZ group. COX-2 immunopositivity was mild in the HG, FUAZ, and LTAZ groups and intense in the ATZ group. (□) indicates the region of interest. Immunoreactivity was graded as follows: − (absent), + (mild), ++ (moderate) and +++ (intense). Scale bar = 50 μm.

**Figure 7:**
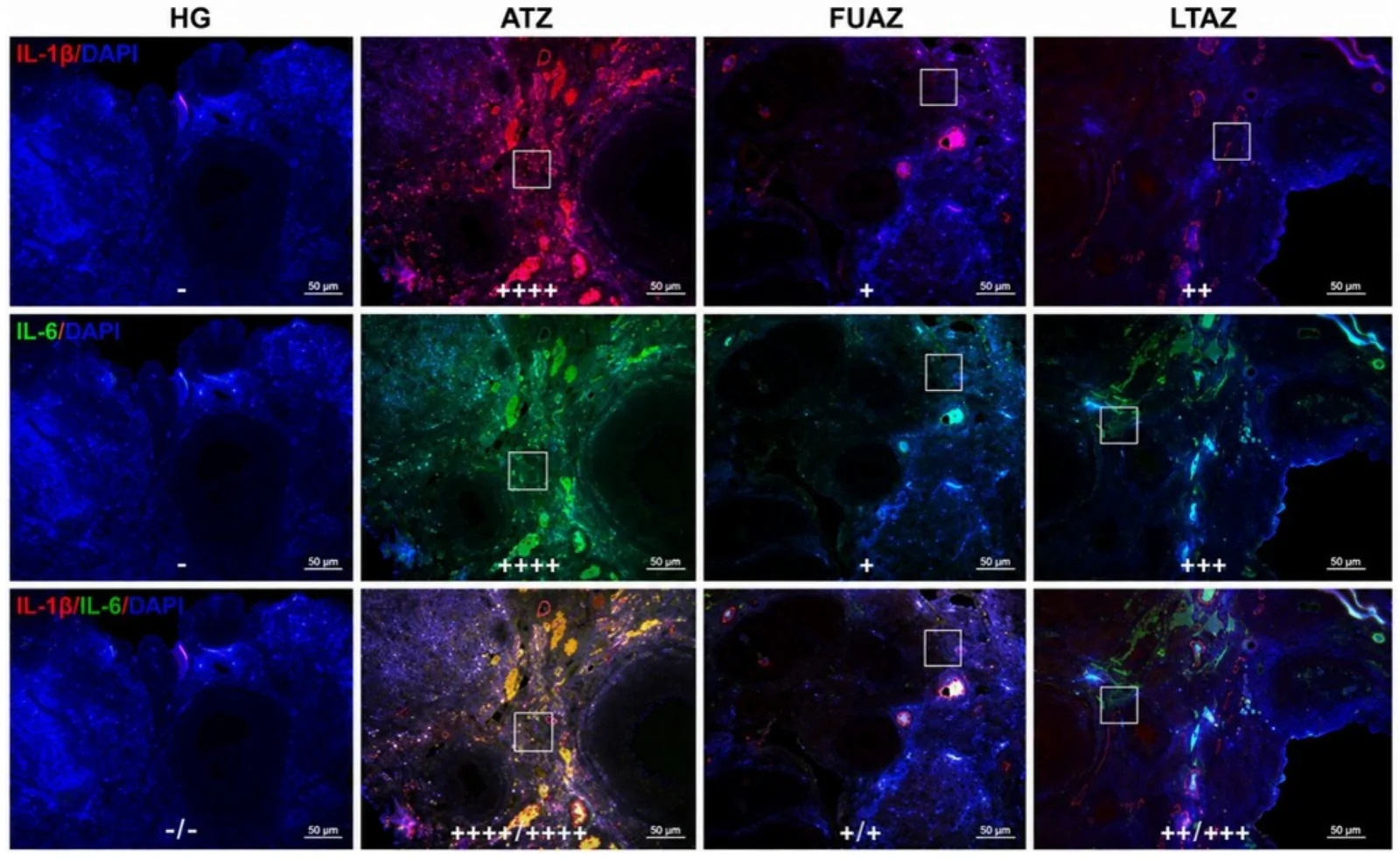
IL-1β immunopositivity was absent in the HG, very intense in the ATZ group, mild in the FUAZ group, and moderate in the LTAZ group. IL-6 immunopositivity was absent in the HG, very intense in the ATZ group, mild in the FUAZ group, and intense in the LTAZ group. (□) indicates the region of interest. Immunoreactivity was graded as follows: − (absent), + (mild), ++ (moderate), +++ (intense), and ++++ (very intense). Scale bar = 50 μm.

**Figure 8:**
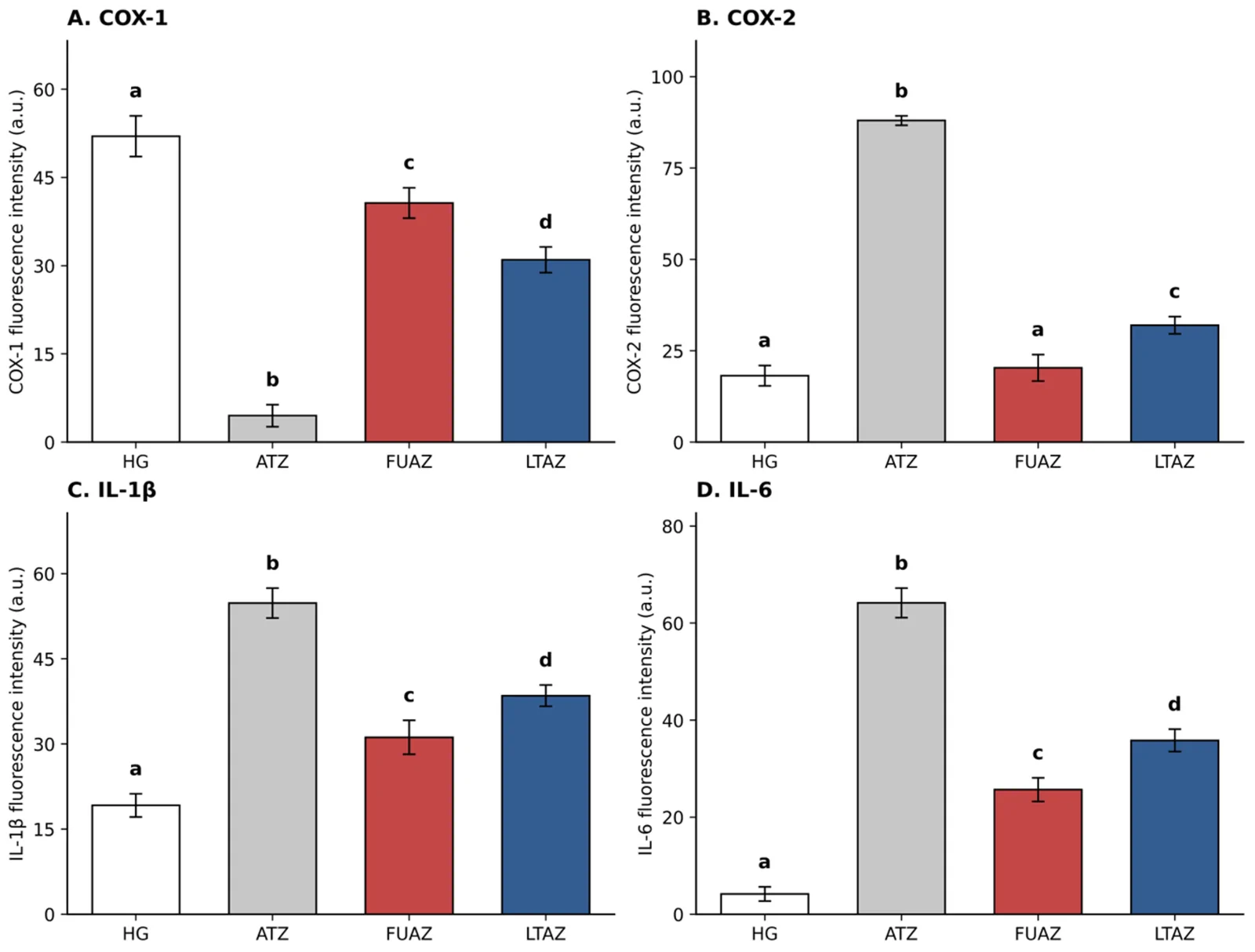
Quantitative analysis of COX-1, COX-2, IL-1β, and IL-6 immunofluorescence intensity in ovarian tissues. (**A**) COX-1, (**B**) COX-2, (**C**) IL-1β, and (**D**) IL-6 immunofluorescence intensities in the healthy group (HG), atezolizumab group (ATZ), flunarizine plus atezolizumab group (FUAZ), and lutein plus atezolizumab group (LTAZ). Data are presented as mean ± standard deviation (SD; *n* = 6 per group) in arbitrary units (a.u.). Different superscript letters indicate statistically significant differences between groups (*p* < 0.05).

Double immunofluorescence staining revealed differences in cyclooxygenase-1 (COX-1), cyclooxygenase-2 (COX-2), IL-1β, and IL-6 immunoreactivity among the experimental groups (Figure 6 and Figure 7). COX-1 immunopositivity was intense in HG, virtually absent in ATZ, moderate in FUAZ, and mild in LTAZ, whereas COX-2 immunopositivity was intense in ATZ and relatively mild in the other groups (Figure 6). IL-1β and IL-6 immunopositivity were virtually absent in HG, markedly increased in ATZ, relatively weak in FUAZ, and moderate in LTAZ (Figure 7). Quantitative ImageJ (version 1.53c) analysis confirmed significant intergroup differences (Figure 8 and Table 2; *p* < 0.05). Compared with HG, ATZ exhibited significantly lower COX-1 and higher COX-2, IL-1β, and IL-6 fluorescence intensities. Both FUAZ and LTAZ showed significantly higher COX-1 and lower COX-2, IL-1β, and IL-6 intensities than ATZ. COX-1 intensity was higher, whereas IL-1β and IL-6 intensities were lower, in FUAZ than in LTAZ. No significant difference in COX-2 intensity was observed between HG and FUAZ. Overall, the quantitative findings were consistent with the representative images, and several fluorescence intensity measurements in FUAZ were closer to HG values than those in LTAZ at the investigated doses.

## 3. Discussion

The present study evaluated atezolizumab-associated ovarian alterations in rats using biochemical, histopathological, and double immunofluorescence analyses. Oxidative stress is an important mechanism of reproductive tissue injury, and MDA is a marker of lipid peroxidation [22,23]. The increased ovarian MDA levels observed in the ATZ group suggest enhanced oxidative stress, consistent with previous reports of elevated MDA levels following atezolizumab administration [24].

The tGSH, SOD, and CAT parameters measured to evaluate the toxic effects of atezolizumab on ovarian tissue are important endogenous antioxidant defense mechanisms that protect cells against oxidative stress [25]. The reductions in tGSH, SOD, and CAT levels observed in the atezolizumab-treated group indicate a weakening of the cellular antioxidant defense system. tGSH, SOD, and CAT are well-established biological markers of oxidative stress [26]. The alterations observed in our study suggest that atezolizumab administration was associated with disruption of the oxidant–antioxidant balance and increased oxidative stress in ovarian tissue.

**Figure 9:**
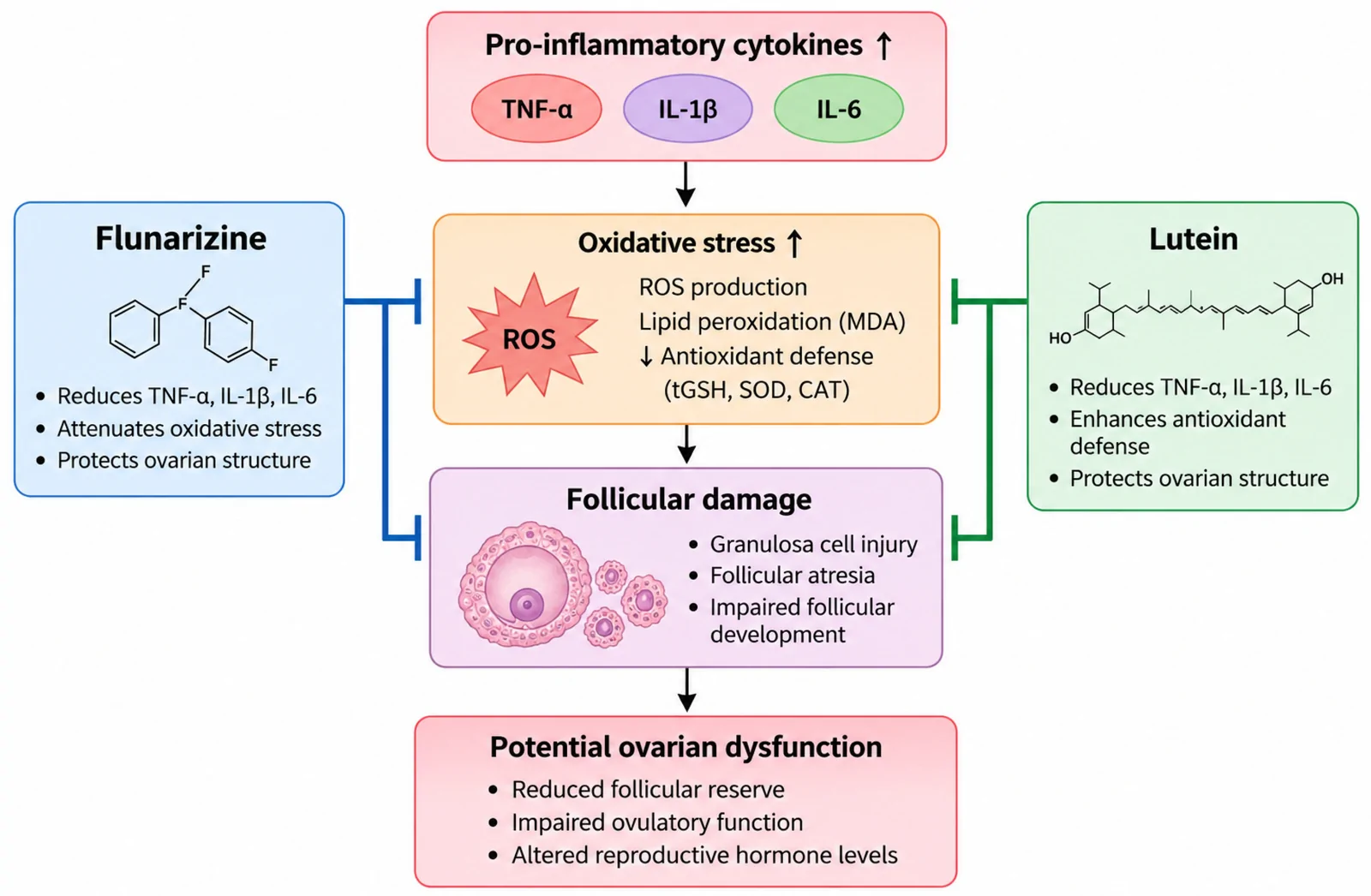
Proposed relationships among pro-inflammatory cytokines, oxidative stress, follicular injury, and potential ovarian dysfunction.

Atezolizumab enhances T-cell activity through PD-L1 blockade in target-appropriate systems [2]. However, functional PD-L1 target engagement was not demonstrated in the present rat model; therefore, this established mechanism cannot be assumed to explain the inflammatory alterations observed in the present study. Accordingly, the mechanism underlying the observed oxidative and inflammatory alterations remains uncertain and cannot be specifically attributed to PD-L1 blockade. In addition, cases of immune-related ovarian insufficiency associated with immune checkpoint inhibitors have been reported in the literature [8]. Therefore, the effects of atezolizumab on ovarian tissue TNF-α and IL-1β levels were also investigated in the present study. TNF-α and IL-1β are well-known pro-inflammatory cytokines [27]. Elevated levels of TNF-α and IL-1β are indicative of inflammatory activation and are closely associated with oxidative stress [28]. In particular, immune checkpoint inhibitors have been shown to increase TNF-α levels in ovarian tissue, potentially contributing to inflammatory ovarian alterations [11]. The biochemical findings obtained in the present study suggest that atezolizumab administration was associated with oxidative stress in ovarian tissue. These results are consistent with previous reports indicating that immune checkpoint inhibitors are associated with toxicity mechanisms involving inflammation, ROS generation, and cellular injury [29]. The potential relationships among pro-inflammatory cytokines, oxidative stress, follicular damage, and ovarian dysfunction are illustrated in Figure 9.

Serum prolactin levels were measured to evaluate hormonal changes associated with atezolizumab administration. Prolactin is involved in reproductive function, and alterations in its circulating levels may affect reproductive physiology [30,31]. However, the biological significance of the observed prolactin changes in the present experimental model remains unclear.

To evaluate hormonal changes associated with atezolizumab administration, serum AMH levels were measured. AMH is produced by ovarian granulosa cells and is widely used as a marker related to follicular activity and ovarian function [32]. In the present study, serum AMH levels were significantly decreased in the ATZ group compared with the HG. Previous studies have also reported decreased AMH levels following gonadotoxic treatments such as chemotherapy and radiotherapy [33]. However, because systematic quantitative follicle counting using serial ovarian sections was not performed, the observed decrease in serum AMH in the present study should not be considered sufficient evidence of a reduction in ovarian reserve.

Flunarizine, which was evaluated in combination with atezolizumab in the present study, is a calcium channel blocker that reduces Ca^2+^ influx and oxidative stress [34,35]. In the present study, the FUAZ group showed lower oxidant-marker values and higher antioxidant-marker values than the ATZ group. Because intracellular Ca^2+^ levels and Ca^2+^-dependent signaling were not assessed, the present findings do not establish whether the calcium-channel-blocking activity of flunarizine contributed to the observed between-group differences. Yavuz et al. reported that flunarizine attenuated erlotinib-induced ovarian damage, accompanied by improvements in oxidative stress and antioxidant parameters in ovarian tissue [36]. Previous experimental findings have demonstrated that flunarizine significantly attenuates increases in the pro-inflammatory cytokines TNF-α, IL-1β, and IL-6 in ovarian tissue, supporting its anti-inflammatory effects [37].

At the specific doses and regimen tested, several biochemical, histopathological, and immunofluorescence outcomes in the FUAZ group were closer to HG values than the corresponding outcomes in the LTAZ group. However, the present study was not designed to establish comparative pharmacological efficacy between flunarizine and lutein. The agents were evaluated at single doses derived from different previous experimental studies, without dose–response assessment, pharmacokinetic exposure measurements, or demonstration of pharmacologically comparable dosing. Therefore, the observed between-group differences should not be interpreted as evidence that flunarizine is intrinsically more effective or superior to lutein.

In the present study, serum AMH levels were significantly lower in the ATZ group than in the HG, consistent with previous reports associating reduced AMH levels with ovarian damage and impaired follicular activity [32,38,39]. Notably, AMH levels were significantly higher in the FUAZ group than in the ATZ group and comparable to those in HG, whereas the LTAZ group did not differ significantly from ATZ. These findings suggest that flunarizine co-administration was associated with a more favorable AMH profile than lutein at the investigated doses. Previous studies have linked reduced AMH levels to oxidative stress [40], which is consistent with the elevated MDA and reduced antioxidant parameters observed in our ATZ group. Conversely, the higher AMH levels in FUAZ coincided with lower MDA and higher tGSH, SOD, and CAT values, suggesting a possible association between improved redox balance and the observed hormonal differences. However, because the AMH assay was not independently validated for rat serum, systematic follicle counting was not performed, and estrous-cycle stages were not monitored, these findings remain exploratory and cannot establish preservation of ovarian reserve or a causal antioxidant mechanism.

Lutein, which was evaluated in combination with atezolizumab in the present study, is a carotenoid with well-established antioxidant and anti-inflammatory properties [16]. In the present study, the LTAZ group showed lower oxidative stress-marker values and higher antioxidant-marker values than the ATZ group. These between-group differences are consistent with, but do not establish, a contribution of the reported antioxidant properties of lutein [41]. Lutein has been shown to reduce oxidative stress by scavenging ROS and suppressing inflammatory responses. It has also been shown to inhibit the production of pro-inflammatory cytokines such as TNF-α and IL-1β [42]. TNF-α and IL-1β levels were also lower in the LTAZ group than in the ATZ group. However, because a lutein-only group was not included, these findings should be interpreted not as definitive evidence of a protective effect specifically attributable to lutein, but rather as between-group differences observed during co-administration. Although lutein treatment was associated with numerical changes in serum prolactin and AMH levels, these differences were not statistically significant compared with the atezolizumab-treated group. Therefore, the present findings do not provide sufficient evidence to conclude that lutein significantly preserved these hormonal parameters. Nevertheless, lutein has been reported to suppress inflammatory responses by reducing oxidative stress and to support reproductive function [43]. Further studies are required to clarify whether these properties may influence endocrine and ovarian functional outcomes.

Our histopathological findings were consistent with the biochemical findings. Follicular degeneration, edema, and hyperemia were more pronounced in the ATZ group than in the HG. However, because estrous-cycle stages were not monitored by vaginal cytology, physiological variability related to cycle stage may have contributed to differences in ovarian morphology among the groups. Therefore, the observed histopathological differences cannot be attributed solely to the experimental treatments. Because systematic follicle counting was not performed, these findings should not be interpreted as a quantitative assessment of ovarian reserve but rather as short-term structural alterations in ovarian tissue. Previous studies have also reported structural and functional alterations in gonadal tissues following ICI exposure [44]. At the specific doses and regimen tested, histopathological scores were lower in the FUAZ and LTAZ groups than in the ATZ group and were lower in the FUAZ group than in the LTAZ group. These findings represent between-group differences and do not establish an independent protective effect or greater intrinsic efficacy of either adjunct. Previous experimental studies have reported favorable histopathological findings with flunarizine in ovarian ischemia–reperfusion injury [37] and effects of lutein on follicular development and oxidative-damage markers [45]; however, these observations were obtained in different experimental models and should not be directly extrapolated to the present setting.

Double immunofluorescence analysis revealed higher COX-2, IL-1β, and IL-6 immunoreactivity and lower COX-1 immunoreactivity in the ATZ group than in the HG. As COX-1 and COX-2 are involved in normal ovarian physiology and their expression varies with reproductive status, these findings should be interpreted cautiously, particularly because estrous-cycle stages were not controlled [46]. Increased IL-1β and IL-6 immunoreactivity in the ATZ group was consistent with inflammatory alterations [28]. Compared with ATZ, both FUAZ and LTAZ exhibited lower COX-2, IL-1β, and IL-6 and higher COX-1 immunoreactivity. Several measurements in FUAZ were closer to HG values than those in LTAZ at the tested doses. However, these differences do not establish superior intrinsic efficacy of flunarizine, independent protective effects of either adjunct, or the mechanism of atezolizumab-associated reproductive toxicity in humans.

This study has several limitations. Although atezolizumab has demonstrated cross-reactivity with rat PD-L1, direct target binding and functional PD-L1 blockade were not confirmed in our model. Therefore, the observed ovarian alterations cannot be specifically attributed to PD-L1 inhibition or extrapolated to human reproductive toxicity. Future studies should use species-appropriate models with verified target engagement, such as humanized PD-L1 models or species-specific surrogate antibodies. Furthermore, the absence of an isotype- or formulation-matched intraperitoneal control prevents differentiation of atezolizumab-specific effects from nonspecific antibody, formulation, or injection-related effects. The lack of flunarizine-only and lutein-only groups precludes conclusions regarding their independent protective effects. Finally, only single doses and a short-term protocol were evaluated, without dose–response or pharmacokinetic assessments. The doses of the two agents were selected from different previous experimental studies and cannot be assumed to be pharmacologically equivalent. Therefore, differences between the FUAZ and LTAZ groups should be interpreted only as between-group differences observed at the specific doses and regimen tested and not as evidence that either agent has greater intrinsic efficacy or is superior to the other. Longitudinal body-weight changes and standardized behavioral or systemic toxicity parameters were also not prospectively assessed. Furthermore, the molecular mechanisms underlying the observed differences were not directly investigated; intracellular Ca^2+^ levels, ATP content, mitochondrial function, and Ca^2+^-dependent or other downstream signaling pathways were not assessed. Therefore, the contribution of calcium-channel blockade to the findings observed in the FUAZ group remains hypothetical and cannot be established from the present data. Serum prolactin and AMH levels were measured as exploratory hormonal parameters; however, ovarian reproductive function was not directly assessed. Accordingly, the hormonal findings should be considered exploratory and should not be assigned substantial mechanistic or functional significance. Estrous-cycle stages were not monitored, systematic quantitative follicle counting using serial ovarian sections was not performed, and mating, ovulation, pregnancy, and other long-term fertility outcomes were not evaluated. Additionally, systematic necropsy examinations and macroscopic documentation of ovarian tissues were not performed, limiting the assessment of potential gross pathological alterations. Given the small sample size (*n* = 6 per group), an uneven distribution of estrous cycle stages may have influenced the observed hormonal, histopathological, and immunofluorescence differences between groups. Therefore, the hormonal and histopathological findings cannot establish changes in ovarian reserve or reproductive protection. Given the multiple outcomes and lack of a prespecified primary endpoint, the analyses remain exploratory. Further studies with verified target engagement, appropriate antibody and adjunct-only controls, dose–response and pharmacokinetic assessments, validated hormonal assays, mechanistic investigations, quantitative follicle counting, and functional reproductive evaluations are needed.

## 4. Materials and Methods

### 4.1. Experimental Animals

A total of 24 female Wistar albino rats (*Rattus norvegicus*), 9–10 weeks old and weighing 255–270 g, were used in this study. All animals were obtained from the Medical Experimental Application and Research Center of Erzincan Binali Yıldırım University. Prior to the experiment, the rats were housed under standard laboratory conditions at a controlled room temperature of 22 ± 2 °C with a 12 h light/12 h dark cycle and were provided with standard pellet chow and water ad libitum. The animals were acclimatized to the laboratory environment for one week before the experimental procedures. All experimental protocols and procedures were approved by the Local Animal Experiments Ethics Committee of Erzincan Binali Yıldırım University (Approval Date: 21 May 2026; Meeting No: 2026/05) and were conducted in accordance with the European Parliament Directive 2010/63/EU and the ARRIVE guidelines [47].

### 4.2. Chemicals

Flunarizine (5 mg tablets) used in the experiment was obtained from Johnson & Johnson Health Care Products Industry and Trade Co., Ltd. (Istanbul, Türkiye). Atezolizumab, in its commercial formulation Tecentriq^®^ (1200 mg/20 mL; F. Hoffmann-La Roche Ltd., Basel, Switzerland), was supplied by the Oncology Clinic of Erzincan Binali Yıldırım University Mengücek Gazi Training and Research Hospital. Lutein was obtained from Solgar (Leonia, NJ, USA), and ketamine was purchased from Pfizer Pharmaceuticals Co., Ltd. (Istanbul, Türkiye).

### 4.3. Experimental Groups

The animals were randomly assigned to four groups (*n* = 6 per group): healthy group (HG), atezolizumab group (ATZ), flunarizine + atezolizumab Group (FUAZ), and lutein + atezolizumab Group (LTAZ). Randomization was performed using a random-number table. No formal a priori sample-size calculation was performed. The sample size was selected based on previous comparable experimental studies. Estrous cycle stages were not monitored or synchronized by vaginal cytology before or during the experimental period. Therefore, potential variations associated with the estrous cycle could not be controlled in the present study.

### 4.4. Experimental Procedure

Flunarizine (5 mg/kg) [37] was administered orally by gavage to the FUAZ group (*n* = 6), while lutein (1 mg/kg) [48] was administered orally by gavage to the LTAZ group (*n* = 6). The oral gavage route was selected to ensure controlled and reproducible delivery of the predefined doses and was consistent with the administration approaches used in the experimental studies on which the selected doses were based [37,48]. The HG (*n* = 6) and ATZ (*n* = 6) groups received distilled water as a vehicle via the same route and method of administration. One hour after the administration of flunarizine, lutein, or vehicle, atezolizumab was injected intraperitoneally (i.p.) at a dose of 10 mg/kg into the FUAZ, LTAZ, and ATZ groups on days 1 and 4. This dosing regimen was selected based on a previous preclinical study in rats in which atezolizumab was administered i.p. at 10 mg/kg on days 1 and 4 [49]. Atezolizumab was withdrawn directly from the commercially available Tecentriq^®^ vial and administered without any further dilution or preparation. The formulation was stored and administered according to the manufacturer’s recommendations. Because of interspecies differences in pharmacokinetics, pharmacodynamics, and systemic exposure, direct comparison of the experimental dose used in rats with the clinical dose is not straightforward. Therefore, the experimental dose used in the present study should not be considered directly equivalent to the clinical dose of atezolizumab. Flunarizine and lutein were administered once daily for six consecutive days.

### 4.5. Blood and Ovarian Tissue Collection

On day 7, the rats were euthanized under high-dose ketamine anesthesia (120 mg/kg), and ovarian tissues were collected for analysis. Blood and ovarian tissue samples were collected for biochemical, hormonal, histopathological, and double immunofluorescence analyses, as described in the following sections.

### 4.6. Tissue Homogenization and Preparation

Ovarian tissue samples were weighed, cut into small pieces, and immediately frozen in liquid nitrogen. The frozen tissues were pulverized using a mortar and pestle and homogenized in phosphate-buffered saline (PBS, pH 7.4; 1:10, *w*/*v*). After vortexing for 10 s, the homogenates were centrifuged at 10,000× *g* for 20 min at 4 °C (Allegra X-30R, Beckman Coulter, Brea, CA, USA). The resulting supernatants were collected and stored at −80 °C until biochemical analysis.

### 4.7. Biochemical and Hormonal Analyses

#### 4.7.1. Tissue MDA, tGSH, SOD, and CAT Analyses

Tissue concentrations of MDA, tGSH, and total SOD activity were quantified using commercially available rat-specific enzyme-linked immunosorbent assay (ELISA) kits (MDA, YLA0029Ra; GSH, YLA0121Ra; SOD, YLA0115Ra; YL Biont Co., Ltd., Shanghai, China) in accordance with the manufacturer’s protocols. Catalase (CAT) activity was measured using the spectrophotometric method described by Góth [50]. Total protein concentrations in ovarian tissue homogenates were determined using the Bradford assay, with absorbance measured at 595 nm [51]. CAT activity was normalized to total protein content and expressed as U/mg protein.

#### 4.7.2. Tissue TNF-α and IL-1β Analyses

Rat-specific ELISA kits supplied by YL Biont Co., Ltd. (China) were used to determine tissue concentrations of TNF-α (Catalog No. YLA0118Ra) and IL-1β (Catalog No. YLA0030Ra). Cytokine measurements were performed using tissue supernatants according to the manufacturer’s instructions.

#### 4.7.3. Serum Prolactin Analysis

Serum prolactin levels were measured in blood samples using an immunoassay analyzer (Abbott Architect, Abbott Laboratories, Abbott Park, IL, USA) and the Architect Prolactin Kit (Ref. No. 7K76-25) according to the manufacturer’s protocol.

#### 4.7.4. Serum Anti-Müllerian Hormone Analysis

Serum anti-Müllerian hormone (AMH) levels were determined using the UNICELL-AMH kit (YHLO Biotech Co., Ltd., Shenzhen, China), which is based on a fluorescence immunochromatographic sandwich assay. The analytical sensitivity and measurement range of the assay were 0.1–25.0 ng/mL. The assay was not independently validated for the rat serum matrix; therefore, the resulting AMH measurements were considered exploratory.

### 4.8. Histopathological Analysis

Ovarian tissue samples were fixed in 10% neutral buffered formalin. Following fixation, the tissues were dehydrated through a graded ethanol series, cleared in xylene, infiltrated with molten paraffin, and embedded in paraffin blocks according to standard histological processing procedures [52]. Sections of 5 μm thickness were cut from the paraffin blocks, deparaffinized, rehydrated through a graded ethanol series, and stained with hematoxylin and eosin (H&E) for histopathological examination. Two blinded pathologists independently evaluated all sections. The severity of follicular degeneration, hyperemia and edema was scored semiquantitatively as absent (0), mild (1), moderate (2), or severe (3) [53]. Three non-consecutive sections per animal were examined, with six randomly selected, non-overlapping fields evaluated per section at ×400 magnification. Each parameter was scored separately, and the median field score was used as the representative value for each animal. The scoring criteria are summarized in Table 3.

### 4.9. Double Immunofluorescence Staining and Quantitative Image Analysis

#### 4.9.1. Double Immunofluorescence Staining

Five-micrometer-thick tissue sections mounted on poly-L-lysine-coated slides were deparaffinized in xylene, rehydrated through graded alcohol series, and washed with phosphate-buffered saline (PBS). For antigen retrieval, the sections were treated with citrate buffer (pH 7.4) at 800 W for two 5-min cycles. After two 10-min washes with PBS, the sections were incubated in PBS containing gelatin and 0.25% Triton X-100 (Sigma Aldritch, St. Louis, MO, USA) for 10 min. Nonspecific binding was blocked with 5% bovine serum albumin (BSA) for 1 h. Following blocking, the sections were incubated overnight at 4 °C with the following pairs of primary antibodies, each diluted 1:200: monoclonal anti-COX-1 (Santa Cruz Biotechnology, Dallas, TX, USA, Cat. No. sc-19998) and polyclonal anti-COX-2 (BT-Lab, Cat. No. BT-AP08027), or monoclonal anti-IL-1β (Santa Cruz Biotechnology, Cat. No. sc-52012) and polyclonal anti-IL-6 (Affbiotech, Cat. No. DF6087). After washing with PBS, the sections were incubated for 45 min with a secondary-antibody cocktail containing equal amounts of anti-mouse Texas Red (AAT Bioquest, Cat. No. 16853) and anti-rabbit FITC (Elabscience, Wuhan, China, Cat. No. E-AB-1014), each diluted 1:100 in 1% BSA. The sections were subsequently washed with PBS and treated with 10 mM CuSO_4_/50 mM NH_4_Cl solution for 10 min. After washing with distilled water, the sections were counterstained with DAPI (4′,6-diamidino-2-phenylindole) and examined using a Zeiss Axiolab 5 microscope (Zeiss, Oberkochen, Germany) equipped with fluorescence illumination, an Axiocam 305 color camera (Zeiss, Oberkochen, Germany), and a Colibri 3 light source system (Zeiss, Oberkochen, Germany).

#### 4.9.2. Quantitative Image Analysis and Negative Controls

For quantitative image analysis, FITC and Texas Red channels were analyzed separately without channel merging using ImageJ software (National Institutes of Health, Bethesda, MD, USA) [54]. All images were acquired using identical microscope settings, including objective magnification, exposure time, and gain, to ensure consistency and comparability of fluorescence intensity measurements [55]. Mean fluorescence intensity (Mean Gray Value) was measured within predefined regions of interest (ROIs) after background subtraction. Six different fields were evaluated for each animal, and the mean fluorescence intensity obtained from these six fields was averaged to generate a single value for each animal for statistical analysis. Thus, the individual animal, rather than the individual microscopic field, was considered the experimental unit (*n* = 6 animals per group). Negative controls were included in each staining run to verify staining specificity and assess nonspecific secondary-antibody binding. For the negative controls, the primary antibodies were omitted and tissue sections were incubated with antibody diluent alone, followed by the same anti-mouse Texas Red and anti-rabbit FITC secondary-antibody cocktail. No meaningful background fluorescence or nonspecific signal was observed in the negative-control sections.

### 4.10. Statistical Analysis

Biochemical data were expressed as mean ± standard deviation (SD). The normality of data distribution was assessed using the Shapiro–Wilk test [56], and homogeneity of variances was evaluated using Levene’s test [57]. Differences among groups were analyzed using one-way analysis of variance (ANOVA), followed by Tukey’s honestly significant difference (HSD) post hoc test for multiple comparisons [58]. Statistical analyses were performed using SPSS for Windows version 18.0 (SPSS Inc., Chicago, IL, USA). A *p*-value of <0.05 was considered statistically significant. Histopathological and immunofluorescence scores were expressed as median (minimum–maximum) values. Differences among groups were analyzed using the Kruskal–Wallis test [59]. When a significant difference was detected, pairwise comparisons were performed using the Mann–Whitney U test with Bonferroni correction [60]. For the six pairwise comparisons among the four groups, a Bonferroni-adjusted *p*-value of <0.0083 was considered statistically significant. Quantitative fluorescence intensity data obtained using ImageJ were expressed as mean ± SD and analyzed using one-way ANOVA followed by Tukey’s HSD post hoc test, with *p* < 0.05 considered statistically significant. The individual animal was considered the experimental unit for all statistical analyses. Microscopic fields and tissue sections were treated as subsamples, and field-level measurements were summarized to obtain a single representative value for each animal before inferential statistical analysis.

## 5. Conclusions

In conclusion, atezolizumab administration was associated with oxidative stress, inflammatory responses, and histopathological alterations in ovarian tissue. Co-administration of flunarizine or lutein was associated with less pronounced alterations in several evaluated parameters, with more favorable findings observed in the flunarizine group at the selected doses. These findings provide preliminary experimental evidence of differences in ovarian biochemical, inflammatory, and histopathological outcomes following co-administration, without establishing independent protective efficacy or causal mechanisms. Further studies incorporating appropriate control groups, validated molecular assessments, and functional reproductive evaluations are needed to clarify the underlying mechanisms and clinical relevance of these findings.

## Figures and Tables

**Table 1:** Histopathological scores of ovarian tissue lesions.

Group	Hyperemia	Edema	Follicular Degeneration
HG	0 (0–0) ^d^	0 (0–0) ^d^	0 (0–0) ^d^
ATZ	3 (3–3) ^a^	3 (2–3) ^a^	3 (2–3) ^a^
FUAZ	1 (1–2) ^b^	1 (1–1) ^b^	1 (1–2) ^b^
LTAZ	2 (1–2) ^c^	2 (2–2) ^c^	2 (2–3) ^c^

Values are expressed as median (min–max). Different superscript letters indicate significant differences between groups according to the Kruskal–Wallis test followed by pairwise Mann–Whitney U tests with Bonferroni correction (adjusted *p* < 0.0083). Abbreviations: HG, healthy group; ATZ, atezolizumab-treated group; FUAZ, flunarizine + atezolizumab-treated group; LTAZ, lutein + atezolizumab-treated group.

**Table 2:** Quantitative fluorescence intensity analysis of COX-1, COX-2, IL-1β, and IL-6 in ovarian tissues.

Group	COX-1 (a.u.)	COX-2 (a.u.)	IL-1β (a.u.)	IL-6 (a.u.)
HG	52.00 ± 3.46 ^a^	18.17 ± 2.78 ^a^	19.17 ± 2.04 ^a^	4.16 ± 1.47 ^a^
ATZ	4.50 ± 1.87 ^b^	88.00 ± 1.27 ^b^	54.83 ± 2.64 ^b^	64.17 ± 3.06 ^b^
FUAZ	40.67 ± 2.58 ^c^	20.33 ± 3.61 ^a^	31.17 ± 2.99 ^c^	25.67 ± 2.42 ^c^
LTAZ	31.00 ± 2.19 ^d^	32.00 ± 2.36 ^c^	38.50 ± 1.87 ^d^	35.83 ± 2.31 ^d^

Values are expressed as mean ± SD (*n* = 6 animals per group). Fluorescence intensity was quantified using ImageJ as background-corrected Mean Gray Value and expressed in arbitrary units (a.u.). Different superscript letters indicate statistically significant differences among groups, as determined by one-way ANOVA followed by Tukey’s HSD post hoc test (*p* < 0.05).

**Table 3:** Histopathological scoring criteria for follicular degeneration, edema, and hyperemia in ovarian tissue.

Score	Severity	Follicular Degeneration	Edema	Hyperemia
0	Absent	Normal follicular architecture with intact granulosa and theca cell layers	No interstitial edema	Normal vascular appearance
1	Mild	Minimal follicular degeneration with slight cellular disorganization	Mild, localized interstitial edema	Mild vascular congestion
2	Moderate	Noticeable follicular degeneration with moderate cellular disorganization and vacuolization	Moderate interstitial edema	Moderate vascular congestion
3	Severe	Extensive follicular degeneration with marked disruption of follicular architecture	Severe, widespread interstitial edema	Marked vascular congestion

Histopathological alterations were graded using a four-point semiquantitative scoring system: 0 (absent), 1 (mild), 2 (moderate), and 3 (severe). Each parameter was evaluated separately according to the morphological criteria described in the table.

## Data Availability

The original contributions presented in this study are included in the article/Supplementary Materials. Further inquiries can be directed to the corresponding author.

## Supplementary Materials

The following supporting information can be downloaded at https://www.mdpi.com/article/10.3390/ijms27219862/s1.

## Abbreviations

The following abbreviations are used in this manuscript:

PD-L1	Programmed death-ligand 1
PD-1	Programmed death-1
ATP	Adenosine triphosphate
ROS	Reactive oxygen species
MDA	Malondialdehyde
tGSH	Total glutathione
SOD	Superoxide dismutase
CAT	Catalase
TNF-α	Tumor necrosis factor-alpha
IL-1β	Interleukin-1 beta
IL-6	Interleukin-6
COX-1	Cyclooxygenase-1
COX-2	Cyclooxygenase-2
AMH	Anti-Müllerian hormone
ICI	Immune checkpoint inhibitor
