# Supplementary material for: Effects of Flunarizine and Lutein on Atezolizumab-Associated Ovarian Alterations in Rats
Source: Int J Mol Sci. 2026 Nov 3;27(21):9862. doi: 10.3390/ijms27219862 (PMC13763632; doi:10.3390/ijms27219862)
Supplement: Supplementary file 1 [file ijms-27-09862-s001.zip › ijms-4666540-supplementary.pdf]

Table S1. Effects of flunarizine and lutein on ovarian tissue MDA, tGSH, SOD, and CAT levels in atezolizumab-treated rats.

Group	MDA (nmol/mg protein)	tGSH (nmol/mg protein)	SOD (U/mg protein)	CAT (U/mg protein)
HG	4.23 ± 0.19^{d}	6.59 ± 0.17^{a}	8.28 ± 0.13^{a}	9.10 ± 0.33^{a}
ATZ	7.42 ± 0.24^{a}	3.73 ± 0.23^{d}	4.85 ± 0.19^{d}	5.51 ± 0.28^{d}
FUAZ	4.82 ± 0.17^{c}	5.91 ± 0.14^{b}	7.17 ± 0.13^{b}	8.47 ± 0.13^{b}
LTAZ	6.23 ± 0.20^{b}	4.73 ± 0.13^{c}	5.83 ± 0.10^{c}	6.69 ± 0.16^{c}

Values are expressed as mean ± SD (n = 6). Different superscript letters within the same column indicate statistically significant differences according to one-way ANOVA followed by Tukey's HSD post hoc test ($p < 0.05$).

Table S2. Effects of flunarizine and lutein on ovarian tissue TNF-α and IL-1β levels in atezolizumab-treated rats.

Group	TNF-α (pg/mg protein)	IL-1β (pg/mg protein)
HG	3.27 ± 0.22^{d}	2.49 ± 0.19^{d}
ATZ	6.33 ± 0.30^{a}	5.63 ± 0.19^{a}
FUAZ	3.90 ± 0.51^{c}	3.14 ± 0.12^{c}
LTAZ	5.13 ± 0.27^{b}	4.67 ± 0.25^{b}

Values are expressed as mean ± SD (n = 6). Different superscript letters within the same column indicate statistically significant differences according to one-way ANOVA followed by Tukey's HSD post hoc test ($p < 0.05$). Abbreviations: HG, healthy group; ATZ, atezolizumab-treated group; FUAZ, flunarizine + atezolizumab-treated group; LTAZ, lutein + atezolizumab-treated group; TNF-α, tumor necrosis factor-alpha; IL-1β, interleukin-1 beta.

Table S3. Effects of flunarizine and lutein on serum prolactin levels in atezolizumab-treated rats.

Group	Prolactin (ng/mL)
HG	18.00 ± 2.61[ab]
ATZ	22.33 ± 4.46[a]
FUAZ	15.83 ± 2.48[b]
LTAZ	18.17 ± 2.64[ab]

Values are expressed as mean ± SD (n = 6). Different superscript letters within the same column indicate statistically significant differences according to one-way ANOVA followed by Tukey's HSD post hoc test ($p < 0.05$).

Table S4. Effects of flunarizine and lutein on serum AMH levels in atezolizumab-treated rats.

Group	AMH (ng/mL)
HG	0.251 ± 0.014[a]
ATZ	0.164 ± 0.024[b]
FUAZ	0.245 ± 0.014[a]
LTAZ	0.187 ± 0.008[b]

Values are expressed as mean ± SD (n = 6). Different superscript letters within the same column indicate statistically significant differences according to one-way ANOVA followed by Tukey's HSD post hoc test ($p < 0.05$).
